# Comparative analysis of miniature inverted–repeat transposable elements (MITEs) and long terminal repeat (LTR) retrotransposons in six *Citrus* species

**DOI:** 10.1186/s12870-019-1757-3

**Published:** 2019-04-15

**Authors:** Yan Liu, Muhammad Tahir ul Qamar, Jia-Wu Feng, Yuduan Ding, Shuo Wang, Guizhi Wu, Lingjun Ke, Qiang Xu, Ling-Ling Chen

**Affiliations:** 1 National Key Laboratory of Crop Genetic Improvement, Huazhong Agricultural University, Wuhan, 430070 People’s Republic of China; 2 Hubei Key Laboratory of Agricultural Bioinformatics, College of Informatics, Huazhong Agricultural University, Wuhan, 430070 People’s Republic of China; 3 Key Laboratory of Horticultural Plant Biology (Ministry of Education), Huazhong Agricultural University, Wuhan, 430070 People’s Republic of China

**Keywords:** Active transposon families, *Citrus*, Genome diversity, Long terminal repeat (LTR), Retrotransposons, Miniature inverted–repeat transposable elements (MITEs)

## Abstract

**Background:**

Miniature inverted-repeat transposable elements (MITEs) and long terminal repeat (LTR) retrotransposons are ubiquitous in plants genomes, and highly important in their evolution and diversity. However, their mechanisms of insertion/amplification and roles in *Citrus* genome’s evolution/diversity are still poorly understood.

**Results:**

To address this knowledge gap, we developed different computational pipelines to analyze, annotate and classify MITEs and LTR retrotransposons in six different sequenced *Citrus* species. We identified 62,010 full-length MITEs from 110 distinguished families. We observed MITEs tend to insert in gene related regions and enriched in promoters. We found that DTM63 is possibly an active *Mutator*-like MITE family in the traceable past and may still be active in *Citrus*. The insertion of MITEs resulted in massive polymorphisms and played an important role in *Citrus* genome diversity and gene structure variations. In addition, 6630 complete LTR retrotransposons and 13,371 solo-LTRs were identified. Among them, 12 LTR lineages separated before the differentiation of mono- and dicotyledonous plants. We observed insertion and deletion of LTR retrotransposons was accomplished with a dynamic balance, and their half-life in *Citrus* was ~ 1.8 million years.

**Conclusions:**

These findings provide insights into MITEs and LTR retrotransposons and their roles in genome diversity in different *Citrus* genomes.

**Electronic supplementary material:**

The online version of this article (10.1186/s12870-019-1757-3) contains supplementary material, which is available to authorized users.

## Background

Miniature inverted-repeat transposable elements (MITEs) are a type of non-autonomous DNA transposons lacking their own transposases [1]. They are widely present in eukaryotes, especially in plant genomes. MITEs have the following characteristics: (1) same as autonomous DNA transposons, MITEs possess inverted repeats flanked by small direct repeats (target site duplication, TSD) and shorter length (usually < 800 bp), (2) some MITEs may transcribe and form double strand RNAs, which may further derive into small RNAs (sRNAs) [2], and (3) MITEs can achieve a very high copy number as compared to other transposons in the host genome [3, 4].

Jiang and colleagues discovered an active MITE *mPing* in rice, and later they found two autonomous DNA transposons *Ping* and *Pong* through homology search using the conserved terminals of *mPing*. They also observed that *Pong* can activate the transposition of *mPing* and named this phenomenon “cross-mobilization” [5]. Later, Yang and colleagues confirmed cross-mobilization hypothesis experimentally and reported that MITEs can also be transposed by autonomous DNA transposons belonging to different families [6]. In addition, several studies observed that MITEs tend to be closer to genetic regions [1, 7] and can regulate gene expression [8]. Some transcripts of MITEs can form hairpin structures, which are further recognized by dicer-like (DCL) and generate small RNAs (sRNAs). This phenomenon is confirmed in *Arabidopsis*, rice and human [9, 10]. MITEs derived sRNAs account for about 1/4 of all the sRNAs in rice genome [2], which indicates an important regulatory role of MITEs in transcription. The insertion and elimination of MITEs can lead to presence and absence polymorphism in host genomes, which is thought to be an important aspect of host genome evolution [11].

Usually, long terminal repeat (LTR) retrotransposons start with 5′-TG-3′ and end with 5′-CA-3′. After integration, there are 4~6 bp TSD flanking their acceptor sites. Generally, autonomous LTR retrotransposons contain two genes in their internal regions: *Gag* and *Pol*. *Gag* encodes a structural protein of virus-like capsid, and *Pol* encodes a polyprotein that includes aspartic proteinase (AP), reverse transcriptase (RT), RNaseH (RH) and integrase (INT). According to the order of RT/INT and the occurrence of envelope protein (ENV), LTR retrotransposons can be further divided into *Copia*, *Gypsy*, *ERV*, *Bel-pao* and *Retrovirus* superfamily [12].

Previous studies indicated that there is a relative balance of LTR retrotransposons insertion and deletion in host genome. The deletion of LTR retrotransposons is mainly caused by unequal homologous recombination [13, 14] and illegitimate recombination [15]. However, intra-element unequal homologous recombination leads to the formation of solo-LTRs, which are structurally identical to the 5′ LTR or 3′ LTR ends of complete LTR retrotransposons [14] and have TSD flanks at their ends. Illegitimate recombination deletes the internal sequence of LTR retrotransposons and forms a shorter sequence of forwarding repeats at the deletion sites. Previous studies have shown that sequences deleted by illegitimate recombination are five folds higher than sequences eliminated by unequal homologous recombination in *Arabidopsis* [15]. LTR retrotransposons amplification was thought to be one of the main drivers that lead to the significant genome size expansion. Studies have shown that even among closely related species, significant genomic size changes can result from amplification of certain LTR retrotransposons families [16]. Hawkins et al. discovered that the specific amplification of *Gorge3* LTR retrotransposons families led to significant differences in the genome size of *Gossypium* [17]. Besides, LTR retrotransposons tend to insert in the enhancers, repressors or promoters of downstream genes, and subsequently regulate the expression of downstream genes [18, 19]. The formation of blood orange is a good example of LTR retrotransposons based regulation of gene expression [20]. LTR retrotransposons also induce chromosome rearrangement and gene translocation [21]. In a phylogenetic study, Du et al. discovered that 5 *Gypsy* lineages and 6 *Copia* lineages had been separated before the divergence of dicot and monocot species [22]. This can be used as an annotation reference and classification marker in following studies, as well as a guide for the refinement of LTR retrotransposons sequences in annotated genomes. In Du’s study, they found that there was a positive correlation between solo-LTR formation and LTR size [22]. Later, El-Baidouri and Panaud found that there was also a stronger positive correlation between solo-LTR formation and the ratio of LTR/INTERNAL [23]. They also found that horizontal transfer of LTR retrotransposons play an important role in genome evolution [23].

*Citrus* is an important source of vitamins for human health and ranks at top among all the fruits. Previous studies have identified transposable elements (TEs) from published *Citrus* genomes [20, 24–27], but differences in the TE annotation pipelines make these results un-comparable. The mechanisms of MITEs and LTR retrotransposons accumulation and amplification, and their roles in *Citrus* genome evolution and diversity remain poorly understood. The publication of different *Citrus* genomes opens the door to compare TEs and investigate their evolution characteristics in genome diversity. In the present study, we investigated the genomes of 6 *Citrus* species: *C. sinensis* (sweet orange, genome size ~ 367 Mb, contig N50 49.89 kb), *Atalantia buxifolia* (Chinese box orange, genome size ~ 370 Mb, contig N50 23.89 kb), *C. grandis* (pummelo, genome size ~ 380 Mb, contig N50 10.62 Mb), *C. ichangensis* (ichang papeda, genome size ~ 391 Mb, contig N50 76.56 kb), *C. medica* (citron, genome size 406 Mb, contig N50 46.50 kb) and *C. clementina* (clementine, genome size ~ 370 Mb, contig N50 115.90 kb) [26–30].

We developed two comprehensive pipelines to annotate and analyze the MITE-related and LTR-related sequences in the above *Citrus* species, and then studied the amplification model of some *Citrus* MITE families and compared the MITE presence and absence polymorphism ratio between sweet orange and the other 5 *Citrus* species. We investigated the MITE relative abundance in different genomic regions and analyzed the role of MITEs in gene structure variations. MITE-derived small RNAs and their relative derived position were also investigated. Using a relatively conserved method, complete LTR retrotransposons and solo-LTRs were annotated, and we investigated the activation of different lineages and families. Relative solo-LTR abundance of different LTR retrotransposons families was also investigated. Furthermore, we studied the distribution of LTR-related sequences in sweet orange and explored the possible factors of solo-LTRs formation.

## Results

### Annotation and classification of MITEs using a MITE-hunter based pipeline

We annotated and classified MITEs using the pipeline shown in Additional file 1: Figure S1. In manual curation of MITEs seed sequences (Additional file 8: Data set S1), we found that terminal regions of many MITEs were absent (terminal absence/presence = 47:5), which suggested the necessity of boundary correction of MITE seeds calculated by MITE-Hunter [31]. All MITE-related sequences were classified into *Mutator* superfamily, *PIF-Harbinger* superfamily, and *hAT* superfamily, and further divided into 110 families. Each family was named as DT(A/M/H)1-n (*hAT* corresponds to DTA, *Mutator* corresponds to DTM, *PIF-Harbinger* corresponds to DTH, and number 1-n corresponds to specific family number). *Mutator* superfamily contained 82 families, *hAT* superfamily contained 20 families, and *PIF-Harbinger* superfamily contained 8 families. In total, 61,980 full-length MITEs were annotated in *Citrus* species, and the average length of MITE-related sequences covered ~ 3% of the total genome sequences (8~11 Mb in different genomes, Table 1). We found that 99.4% of the full-length MITEs were shorter than 800 bp, and the average length was ~ 302 bp. The average full-lengths of *Mutator* superfamily, *PIF-harbinger* superfamily and *hAT* superfamily were 256, 431 and 563 bp, respectively. Full-length MITEs of *hAT* superfamily were significantly longer than the other two super-families (Wilcox test, *p*-value < 0.001), and the average length of *Mutator* superfamily was the shortest. We also observed that the copies of top 10% full-length MITE families accounted for over 50% of the total full-length MITE copies.

**Table 1:** Comparative statistics of 61,980 full-length MITEs annotated in Citrus species

Species	Copy number	Full-length copy number	Length (Mb)	Content (%)
*Atalantia buxifolia*	47,856	10,095	9.10	2.90%
*Citrus sinensis*	40,886	9529	8.56	2.81%
*Citrus clementina*	36,950	9089	8.02	2.69%
*Citrus medica*	48,872	12,048	10.79	2.84%
*Citrus ichangensis*	46,421	10,984	9.70	2.84%
*Citrus grandis*	39,963	10,235	8.76	2.51%

### One round amplification burst dominated *Citrus* MITEs

**Fig. 1:**
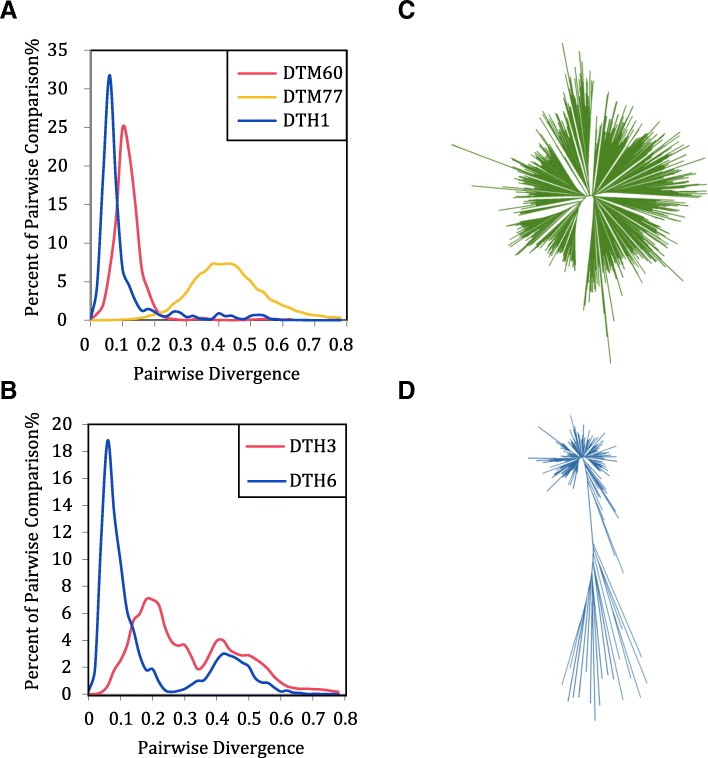
Statistics and phylogenetic trees of MITEs. **a** Unimodal distribution of pairwise divergence among some representative families of full-length MITEs. **b** Bimodal distribution of pairwise divergence among other representative families of full-length MITEs. **c** Phylogenetic tree of DTM77. **d** Phylogenetic tree of DTH6

It is reported that MITE families in rice mainly experienced one or more round of amplification [2]. Using a similar approach in our study, we compared the pairwise divergence distributions in *Citrus*. We found that unimodal distribution of MITE families was dominated in *Citrus*, such as DTM60, DTM77 and DTH1 (Fig. 1a), whereas a few MITE families, including DTH3 and DTH6, showed a bimodal or multimodal distribution (Fig. 1b). We observed that the phylogenetic trees of MITE families with unimodal distribution showed a star shape (Fig. 1c), while those with bimodal or multimodal distributions showed two or multiple clades Fig. 1d). Taken together, MITE families with unimodal distribution mainly experienced one round amplification from a copy of the same MITE or a significant amplification of a close copy of the same family at certain time. While those with bimodal or multimodal distribution mainly experienced two or multi rounds amplification, originated from the amplification events of different members of the same MITE family at different time points.

### Ancient active MITE families and possible active MITE family in trackable past

**Fig. 2:**
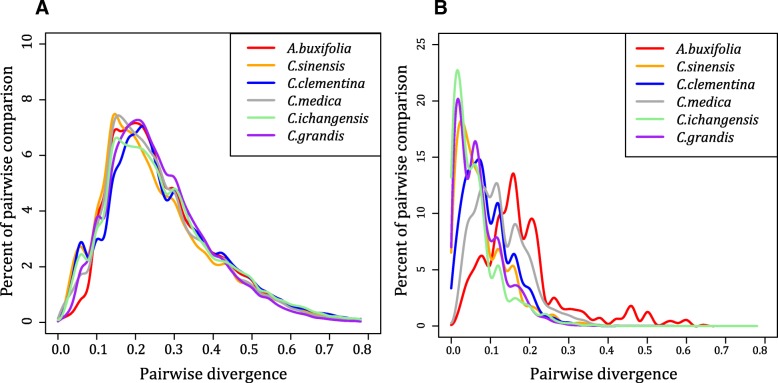
Ancient active MITE family DTM53 and an active MITE family in trackable past DTM63. **a** Pairwise divergence among full-length MITEs of DTM53. **b** Pairwise divergence among full-length MITEs of DTM63

DTM53 has over 1000 full-length copies in each of the six *Citrus* species, their distribution of pairwise divergence is very similar, which are all unimodal curves with mean pairwise divergence of about 0.26 (Fig. 2a). It responded to the divergence time of ~ 23 million years, which is before the divergence of *Citrus* and *Atalantia* genus*.* Similarly, DTM58 (mean pairwise divergence 0.45) and DTM77 (mean pairwise divergence 0.41) also experienced amplification burst before the divergence of *Citrus* and *Atalantia* genus. However, the copy numbers of full-length DTM63 in *A. buxifolia*, *C. sinensis*, *C. clementina*, *C. ichangensis, C. medica* and *C. grandis* are 48, 302, 201, 323, 174 and 496 respectively, and the large difference of DTM63 number indicates the recent amplification of DTM63 family. There were some very similar or identical copies in *C. sinensis*, *C. clementina*, *C. ichangensis* and *C. grandis.* The pairwise divergence of DTM63 showing a peak around the origin (Fig. 2b), indicated that DTM63 may be still an active MITE family in these four species and further research is required for confirmation. The discovered active MITEs mainly came from *Tc1/mariner* and *PIF/Harbinger* super-families, and DTM63 could be the first discovered active MITE family which belonging to *Mutator* superfamily. As DTM63 lacks transposase, its transposition might rely on the autonomous DNA transposons. To figure out the autonomous DNA transposons which activate the transposition of DTM63, we annotated the candidate autonomous DNA transposons and obtained 572 and 585 candidate regions in *C. sinensis* and *C. clementine* respectively. Subsequently, we scanned the candidate regions with the conserved 7 bp terminals and 9~10 bp TSD, however we had not found any *Mutator*-like autonomous DNA transposons which keep the same end sequence as DTM63.

### P/A polymorphism ratios between different *Citrus* genomes reflect genetic distance

MITE insertion and deletion inevitably cause presence or absence (P/A) polymorphism in host genomes [2]. Taken sweet orange as the reference genome, we calculated the P/A polymorphism ratio between sweet orange and the other five *Citrus* species (Table 2). We found that the P/A polymorphism ratio between sweet orange and the primitive *Citrus* variety *A. buxifolia* is 1.3~2.2 times that of the other four species. Therefore, MITE P/A polymorphism has strong correlation with phylogenetic relationships, as the phylogenetic relationship from the closest to farthest to sweet orange is: *C. clementina* (polymorphism ratio 38.38%), *C. grandis* (46.70%)*, C. ichangensis* (59.89%), *C. medica* (65.79%) and *A. buxifolia* (84.49%) respectively*.* The above results indicate that the P/A polymorphism ratio of MITEs reflects the genetic relationship among different *Citrus* species.

**Table 2:** Statistics of MITE P/A polymorphism ratio between sweet orange and the other five *Citrus* species

Feature	*Atalantia buxifolia*	*Citrus clementina*	*Citrus medica*	*Citrus ichangensis*	*Citrus grandis*
Common loci	7571	9233	9727	9324	9745
Absence in *C. sinensis*	3268	1787	3982	3081	2442
Absence in other genomes	3129	1757	2417	2503	2109
Commonly present collinear region loci	1174	5689	3328	3740	5194
Polymorphic ratio (%)	84.49	38.38	65.79	59.89	46.70

### MITEs preferentially inserted in gene flanking regions and play important role in genome diversity

**Fig. 3:**
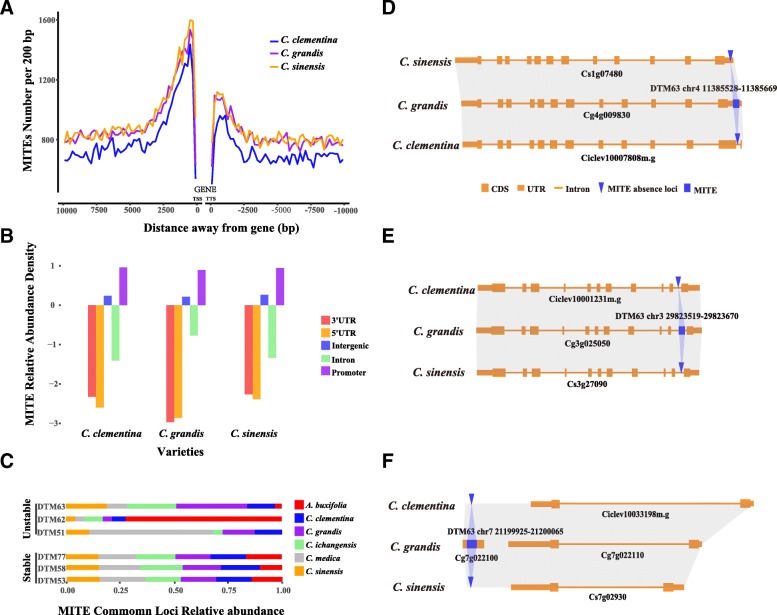
MITEs preferentially inserted in gene flanking regions and play important roles in genome diversity. **a** The distribution of MITEs inserted in upstream and downstream of gene regions in three *Citrus* species. **b** The relative abundance of MITEs in different genomic regions. **c** The stable and unstable MITEs in 6 *Citrus* species. **d** A MITE uniquely inserted into 5′ UTR of *C. grandis* genome. **e** A MITE uniquely inserted into intron of *C. grandis* genome. **f** A MITE uniquely inserted into *C. grandis* genome, and a gene is annotated in the insertion region. (**d**-**f**: Gray shading represents collinear regions. Blue shading represents collinear MITE regions. Orange large rectangles, small rectangles and lines respectively represent CDS, UTR and intron. Blue rectangles and inverted triangle respectively represent MITE and MITE absence loci)

To study whether MITEs favorably inserted in the gene related regions, we calculated the distribution of MITEs insertion in gene regions (from transcription start sites (TSS) to transcription termination sites (TTS)), upstream and downstream of gene regions in sweet orange, pummelo and clementine. We observed that the MITEs insertion distribution patterns were very similar in the three *Citrus* species, and different peaks were observed within 1 kb of upstream and downstream gene regions respectively (Fig. 3a), indicating that MITEs are preferentially inserted in gene flanking regions. Then we analyzed the distribution of MITEs in different genomic regions, including 5′ and 3′ untranslated regions (UTRs), introns, promoters (defined as 1 kb upstream of TSS) and intergenic regions. Considering that most of the genomic regions are intergenic regions, we calculated the relative abundance of MITEs, and observed that the relative density of MITEs was the most abundant in promoter regions, and the least abundant in gene regions (5′ and 3′ UTRs, introns, Fig. 3b). Therefore, MITEs preferentially inserted in gene flanking regions especially in promoters, indicating a *cis*-regulatory role of MITEs for their downstream genes [32, 33].

We compared the copies of different MITE families to investigate their distribution pattern and role in *Citrus* genome evolution, the abundance of different MITE families in six *Citrus* genomes was shown in Additional file 3: Figure S3. There were two types of MITEs sequence distribution pattern. Stable genomic distribution pattern indicated MITE families were similar with almost same copy number in different *Citrus* species, such as DTM53, DTM58 and DTM77. These MITE families experienced amplification burst before the speciation of *Citrus* and *Atalantia* with very few recent MITE insertions or deletions. However, unstable genomic distribution of MITEs families showed obvious distribution bias across different species. For example, the ratios of DTM63 in *C. grandis*, *C. ichangensis* and *C. sinensis* were much higher than *C. medica* and *A. buxifolia* (Fig. 3c), owning to recent transpositions. DTM62 had more copies in *A. buxifolia* than the other 5 *Citrus* species, and DTM51 had more copies in *C. media* than the others. Comparing with stable MITE families, unstable MITE families contributed more divergence to *Citrus* and might play an important role in the diversity of *Citrus* genome*.*

We defined MITEs present in all species as conserved MITEs and the other as non-conserved. The proportion of conserved MITEs inserted into promotor (18.13%) and 5′/3′ UTR regions (1.72%) was lower than non-conserved MITEs (25.76% in promotors and 4.31% in UTRs), which indicated a strong selection effect on MITEs insertion in these regions (Additional file 5: Table S1). Interestingly, newly-inserted MITEs tended to be abundant in promotor regions (26.08% in *C. clementina,* 27.17% in *C. sinensis* and 26.63% in *C. grandis*) comparing to the ancient inserted MITEs (23.97% in *C. clementina,* 25.21% in *C. sinensis* and 23.69% in *C. grandis*) (Additional file 6: Table S2). Figure 3d showed a MITE uniquely inserted into 5′ UTR of *C. grandis*, Fig. 3e showed an example of MITE inserted into intron of *C. grandis* and the MITE insertion in intron did not alter the gene structure, and Fig. 3f showed a MITE inserted into *C. grandis* and a gene was annotated in the insertion region, while there were no genes annotated in the collinear region of *C. clementina* and *C. sinensis* genomes respectively. The above examples indicate that MITEs play important roles in genome structure variations in *Citrus* genomes.

### MITE-derived sRNAs were predominantly derived from MITEs terminals

**Fig. 4:**
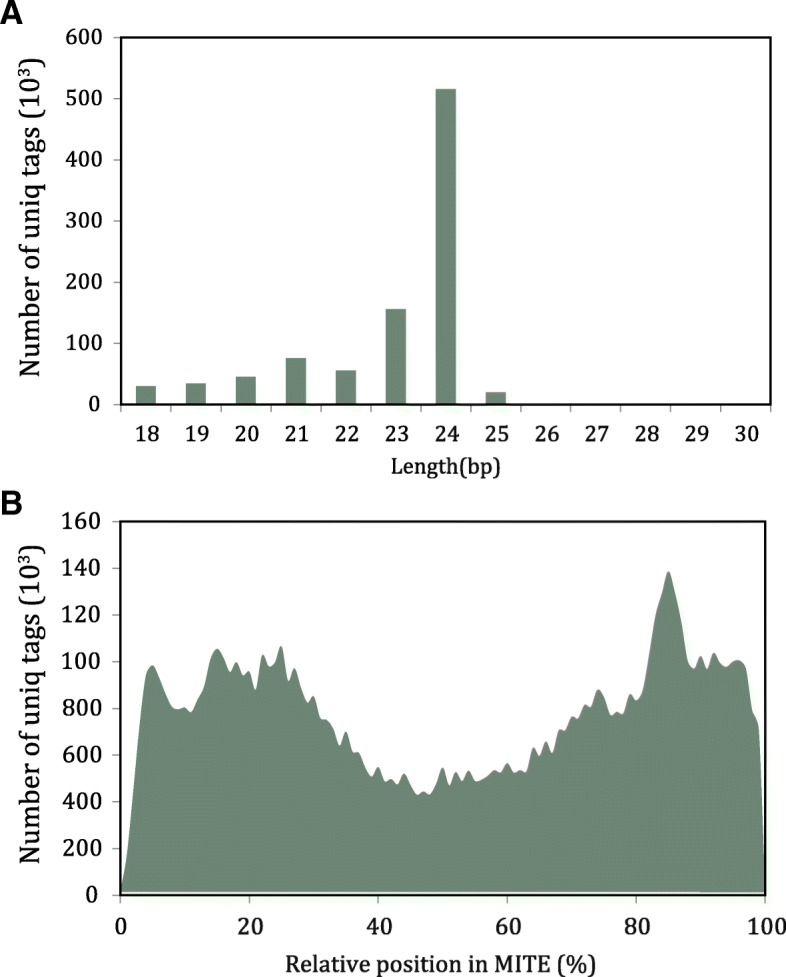
Distribution of MITE-derived small RNAs. **a** Length distribution of MITE-derived small RNAs. **b** The relative position distribution of MITE-derived small RNAs

We collected a total of 14,664,233 unique sRNA tags from previous studies, among them 7,258,262 tags could be aligned to the sweet orange reference genome, and 935,213 tags could be aligned to MITE-related sequences. While filtering out the unmapped tags, MITE-derived sRNAs accounted for 12.9% of the total amount. By looking into the length distribution of MITE-derived sRNAs, we observed that they are predominately 24 bp (Fig. 4a) and derived from different positions of MITEs (Fig. 4b). In comparison with the relative positions from where sRNAs were derived, we found that their distribution in sweet orange is different from the rice [2]. There were only two peaks at both ends with a valley in the middle, indicating that the middle of MITEs derived less sRNAs than the other region in sweet orange, whereas there was another peak in the middle in rice [2].

### LTR retrotransposons annotation, classification and characterization

**Fig. 5:**
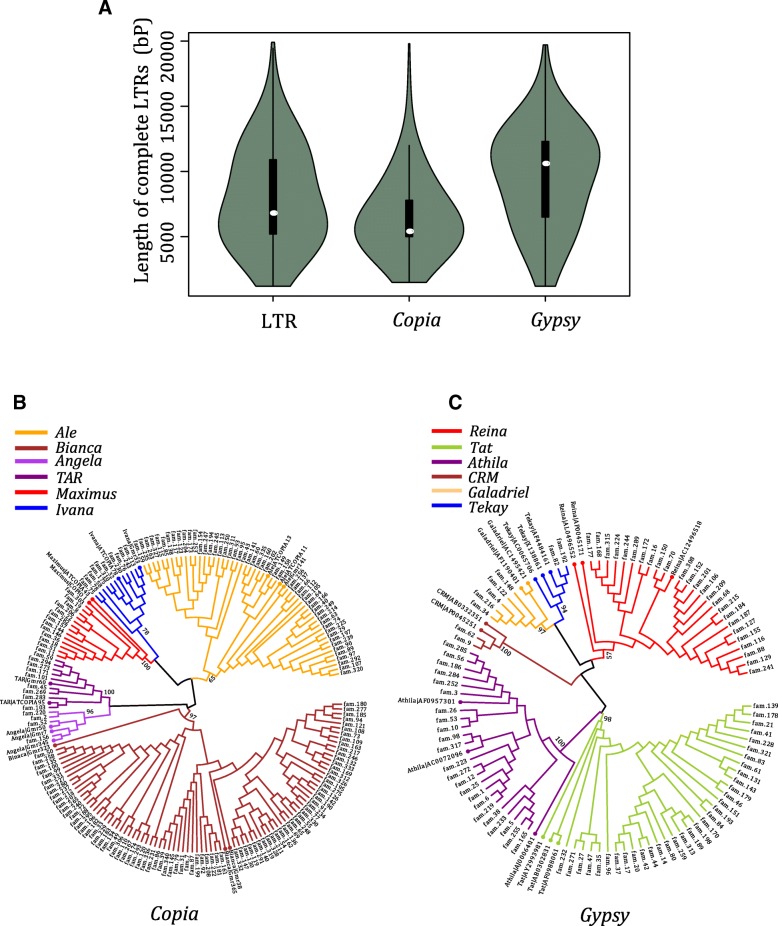
Length distribution and phylogenetic trees of LTRs. **a** Length distribution of complete LTRs (green whiskers and black boxes represent average length distribution, and white circles represent median). **b** *Copia*-like superfamily RT domain phylogenetic tree. **c** *Gypsy*-like superfamily RT domain phylogenetic tree. *Citrus* also keeps six *Gypsy* lineages (reference LTR retrotransposons are shown as italic with circles on branches, others are LTR families in *Citrus*)

Although Wicker and colleagues proposed the “80–80-80” rule for TEs family classification [34], it is still controversial in different LTR studies. In a recent study, it is suggested that use of another cutoff (60% identity and 70% coverage) is more appropriate for the Uwum family in *Zea mays* and RLC_Gmr6/18 family in *soybean* [23]. Considering that both the complete LTR retrotransposons and solo-LTRs have an intact LTR, a cutoff of 75% identity was chosen to classify all LTR retrotransposons into different *Citrus* families. Totally, we obtained 13,371 solo-LTRs and 6670 complete LTR retrotransposons from 340 families (Table 3; Additional file 9: Data set S2). The number of solo-LTRs was roughly equivalent in the 6 species except for *Atalantia buxifolia* and *C. sinensis*, whereas complete LTR retrotransposons varies from 392 to 1904. Considering that the completeness of *C. grandis* is much better than other genomes, its assembly quality for complete LTR retrotransposon regions would be much better than the other five genomes. In addition, the strategy we used might miss some low-copy LTR retrotransposons families of *A. buxifolia*. In this way, *C. grandis* would be a more appropriate choice for further investigation. There were two distinct peaks in the length distribution of complete LTR retrotransposons (Fig. 5a), one peak was around ~ 5.4 kb and the other ~ 12.4 kb, which corresponded to the peaks of *Copia* and *Gypsy* super-families, respectively. The average length of complete LTR retrotransposons in *Copia* superfamily was ~ 6.5 kb, and the average length of complete LTR retrotransposons in *Gypsy* superfamily was ~ 9.3 kb. Consistent with previous studies, *Gypsy* LTR retrotransposons were significantly longer than *Copia* LTR retrotransposons (Wilcox test *p*-value < 0.001) [22, 23, 26, 35, 36]. The ratio of *Gypsy*-like elements to *Copia*-like elements was 1.5 in *C. grandis*, which is slightly higher than in soybean (1.4) [22], but much lower than in rice (4.9) [35] and sorghum (3.7) [36]. In addition, the ratio of solo-LTR to complete LTR retrotransposons was 1.4, which is higher than that in maize, soybean and rice [23].

**Table 3:** Comparative statistics of 13,371 solo-LTRs and 6670 complete LTR retrotransposons

Species	Complete LTR	Solo-LTR	LTR content(%)
*Atalantia buxifolia*	392	1417	20.66
*Citrus sinensis*	487	1946	24.81
*Citrus clementina*	1742	2498	29.31
*Citrus medica*	1205	2389	32.18
*Citrus ichangensis*	940	2416	23.87
*Citrus grandis*	1904	2705	28.32

By calculating the distribution of LTR retrotransposons in sweet orange genome, we found that different from the relatively uniform genomic distribution of MITEs, the distribution of LTR retrotransposons was quite heterogeneous. There were significant peaks in centromere-proximal regions along different chromosomes, which is consistent with the previous studies of other species [37]. A possible explanation for this phenomenon is that, centromere-proximal regions are recombination-suppressed, which leads to the suppression of unequal homology recombination and illegitimate recombination, therefore, LTR retrotransposons in centromere-proximal regions are accumulated.

### Twelve conserved LTR retrotransposon lineages are present in *Citrus*

In the previous studies, the *Copia*-like superfamily was grouped into 6 lineages, *Angela*, *Ale*, *Bianca*, *Ivana*, *Maximus* and *TAR* [12, 38, 39], while *Gypsy*-like superfamily was further grouped into another 6 lineages, *Tekay*, *Galadriel*, *CRM*, *Reina*, *Athila* and *Tat* [40]. Among these, *Tekay, Galadriel, CRM* and *Reina* were *Chromovirus* clades*.* These 12 lineages were separated before the divergence of dicot and monocot species. We retrieved the sequences of RT domains from public data and the LTR retrotransposons identified in this study, including 91 *Gypsy*-like RT and 174 *Copia*-like RT sequences. Then we constructed the phylogenetic trees of RT domain (Fig. 5b, c). From the phylogenetic trees, it was observed that all the 12 lineages were identified in *Citrus*. In addition, the branches of the corresponding families were classified according to the topological structure of the phylogenetic tree.

To investigate the activation and status of these 12 lineages, we counted the family and element numbers of each lineage (Table 4). In *Gypsy*-like superfamily, *Tat* had the most families (33), and *Athila* contained the maximum number of complete LTR retrotransposons (1754). However, in *Copia*-like superfamily, *Bianca* had the most families (86) and complete LTR retrotransposons (1071), while *Angela* had the least families (5) and *TAR* contained the least complete LTR retrotransposons (96).

**Table 4:** Statistics of LTR retrotransposons in 12 conserved citrus lineages

Lineages	Number of families	Number of Complete LTRs	Average Complete LTR length (bp)	Number of solo-LTRs	Average solo-LTR length (bp)
*Ale*	51	637	5259	219	267
*Ivana*	10	155	5574	132	343
*Maximus*	13	439	10,070	1542	1319
*TAR*	10	96	6208	178	750
*Angela*	5	277	8689	1313	1537
*Bianca*	86	1071	5832	961	585
*Reina*	25	309	4712	357	394
*Tekay*	2	26	9068	326	2187
*Galadriel*	5	193	5416	317	473
*CRM*	2	109	8054	605	940
*Athila*	23	1754	11,324	4435	1302
*Tat*	33	732	10,211	1180	838

We further calculated the average length of complete LTR retrotransposons and solo-LTRs for each lineage. Complete LTR retrotransposons of different *Copia* and *Gypsy* lineages showed significant length difference (Kruskal-Wallis rank sum test, *p*-value < 0.001), and solo-LTRs also showed significant length difference (Kruskal-Wallis rank sum test, *p*-value < 0.001). The *Maximus* lineage had an average length of ~ 10 kb, which is the longest in complete *Copia* LTR retrotransposons. Compared to a previous study, the average length of the *Copia* lineages in *Citrus* was roughly equivalent, although *Bianca* has more families and complete LTR retrotransposon members in *Citrus* than in rice, *Arabidopsis* and *Triticeae* [12].

### A few LTR retrotransposons families were active in trackable past and play an import role in *Citrus* genome diversity

**Fig. 6:**
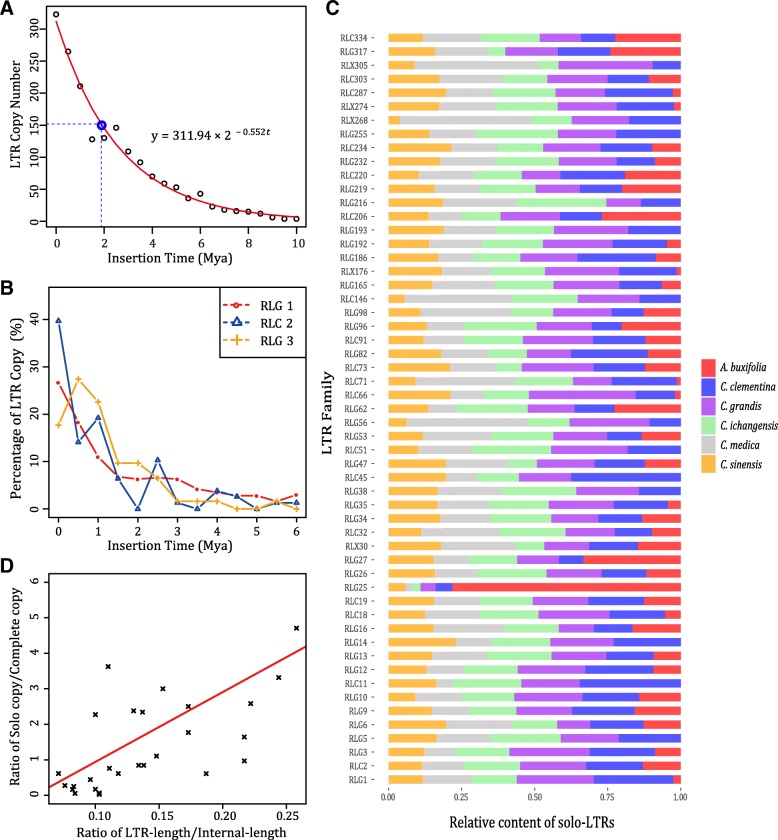
Insertion time and formation factor of LTR. **a** LTR insertion time distribution. **b** Insertion time distribution of three LTR families. **c** Relative content of solo-LTRs in six *Citrus* genomes. Some LTR families keep relative stable like RLC2 and RLG5, while other LTR families shows unstable and may become more active in one specie like RLG25. **d** Solo-LTR formation factor. The ratio of LTR-length/Internal-length shows clear positive correlation with the ratio of Solo copy/Complete copy

Generally, LTR retrotransposons constantly inserted and eliminated in a long-term cycle and maintain the host genome size in a dynamic balance. Through computing the insertion time of LTR retrotransposons, we obtained the LTR retrotransposons insertion time curve (Fig. 6a) and found that the LTR retrotransposons insertion time followed an exponential distribution and their half-life in *Citrus* was ~ 1.8 million years. Meanwhile, we noticed that only a few LTR retrotransposons families were active in trackable past, which were consistent with the previous studies [23]. In *C. grandis*, only eight families, i.e., RLG1, RLG2, RLG3, RLG4, RLG5, RLC7, RLG9 and RLG12 contained more than 30 complete LTR retrotransposons, and the member of RLG1 (476) were larger than the total members (322) of the other seven families. In addition, LTR retrotransposon families with the most copy numbers of complete LTR retrotransposons (such as RLG1, RLG2, RLG3) were usually active recently (Fig. 6b), which indicated that ancient LTR retrotransposons were rapidly removed from the genome by unequal homology recombination and illegitimate recombination. Although copies of complete LTR retrotransposons were highly dependent on the genome integrity, however the copies of solo-LTRs showed less dependency (Table 4). Thus, we compared the copies of different LTR retrotransposons families to investigate whether solo-LTRs played any roles in *Citrus* genome diversity (Fig. 6c). Some families (such as RLC2, RLG5 and RLG16) have similar number of copies among the 6 *Citrus* genomes, indicating these LTR retrotransposons were less active and divergent among different *Citrus* species, we called them stable LTR families. In contrast, some unstable LTR families showed distinct composition among 6 *Citrus* species. For example, 136 solo-LTRs were found belonging to RLG25 in *A. buxifolia*, but in each of the other 5 *Citrus* genomes there were less than 10 copies. Above finding suggested that RLG25 might be more active in *A. buxifolia* and more copies of solo-LTRs had been accumulated through unequal recombination than the other 5 *Citrus* species.

### Insertion time might contribute to solo-LTRs formation

To avoid miss annotation of solo-LTRs which mainly formed by the internal unbalanced homologous recombination of complete LTR retrotransposons and truncated LTR retrotransposons, we treated a region as a solo-LTR only if there were two 4~6 bp exact TSD flanks around. Different from previous studies, only complete LTR retrotransposons were adopted, because LTR retrotransposons without TSD may be the consequence of assembly error, boundary annotation error and inter-element unequal recombination which was shown to be rare in *Arabidopsis* [15]. We took the value of S/C (solo-LTR/complete LTR) to evaluate the relationship between solo-LTR formation and some relative factors (Fig. 6d). Our result revealed a significant correlation between S/C and LTR retrotransposons insertion time (Spearman’s rank correlation *r* = 0.455, *p*-value < 0.01), which showed disagreement with the result in soybean [22]. Similar significant correlation was detected between S/C and LTR size (Spearman’s rank correlation *r* = 0.627, *p*-value < 0.01). We confirmed that there was a strong correlation between S/C and the ratio of LTR/INTERNAL (Spearman’s rank correlation *r* = 0.691, *p*-value < 0.01), which agrees with El-Baidouri’s study [23]. Taken together, we identified that solo-LTRs formation was related with several factors, and longer LTR will favor more stable pair of 5′ and 3′ LTR if they are not too distant, and that the longer the insertion time of LTR retrotransposons, the higher possibility of the unequal recombination will be.

## Discussion

### Annotation and comparison of MITEs and LTR retrotransposons from multiple genomes provides a new insight into *Citrus* evolution and genome diversity

Although a few studies have revealed the important roles of MITEs and LTR retrotransposons in *Citrus* [20, 24, 27], their pivotal role in *Citrus* evolution and diversity has long been overlooked. This is the first study focusing on MITEs and LTR retrotransposons annotation and comparison in *Citrus* to investigate their role especially in genome diversity and evolution. Annotation of MITEs and LTR retrotransposons provides a useful resource for researchers who are interested in *Citrus* MITEs and LTR retrotransposons.

The insertion of MITEs in the *Citrus* genome leads to massive polymorphism, where the inter-genus polymorphism ratio of MITE Insertion Polymorphism (MIP) is much higher than intra-genus, which reflects the genetic relationships among different species. Comparison of MITEs and LTR retrotransposons reveals that some MITEs and LTR retrotransposons are relatively stable in *Citrus* genomes, which shows little composition divergence among *Citrus* genomes, such as DTM53 and RLC2. In contrast, some MITEs and LTR retrotransposons are quite unstable, such as DTM63 and RLG25, they greatly reshape the *Citrus* genomes and some MITEs even play a role in gene structure variations. It remains to be seen that whether other plants show similar MIP among different species and share the same MITEs and LTR retrotransposons stable and unstable composition patterns.

### MITEs and LTR retrotransposons: similarities and difference

**Fig. 7:**
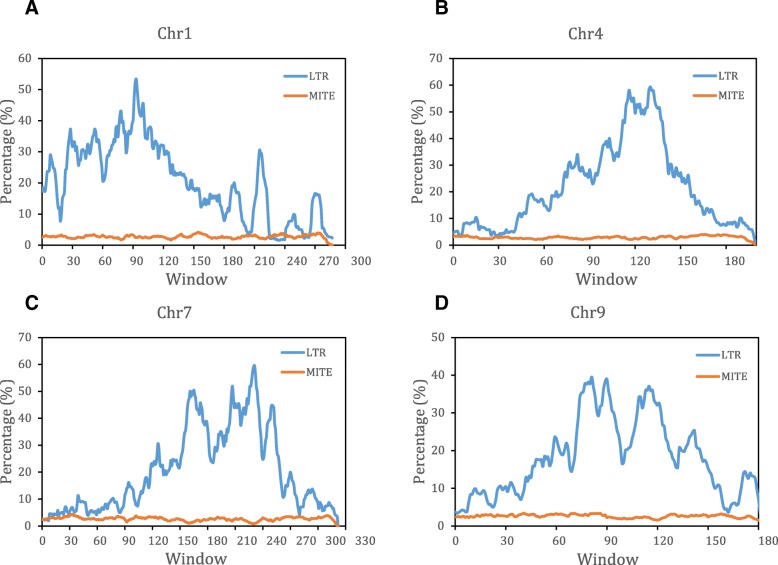
LTR-RTs and MITEs distribution on some representative chromosomes (window size = 1 Mb; step size = 100 kb). **a** LTR retrotransposons and MITEs distribution on chromosome 1. **b** LTR retrotransposons and MITEs distribution on chromosome 4. **c** LTR retrotransposons and MITEs distribution on chromosome 7. **d** LTR retrotransposons and MITEs distribution on chromosome 9

MITEs and LTR retrotransposons are the most important types of transposons and highly characterized in plant genomes. MITEs account for 2.51 to 2.90%, while LTR retrotransposons account for 20.66 to 29.31% in 6 different *Citrus* species. Generally, the average length of LTR retrotransposons is much longer than that of MITEs and account for the most majority part of genome. Both MITEs and LTR retrotransposons are dominated by a few families of full-length (complete) copies. However, the distribution of the two types of transposable elements is quite different, LTR retrotransposons related sequences are enriched in centromere-proximal regions, whereas MITE-related sequences are distributed relatively even across the chromosomes (Fig. 7a-d). Both MITEs and LTR retrotransposons can insert into promoter regions to regulate downstream gene expression as revealed by some previous studies [20, 27].

### DTM63 was active in the trackable past and plays a role in gene-structure variation

The large difference of DTM63 copy number in 6 *Citrus* species identified in this study as well as DTM63 new insertions in citrus bud mutant discovered by Ke et al. [41] indicates that DTM63 is possibly be an active *Mutator*-like MITE family in the trackable past and may still be active in *Citrus*. The average length of DTM63 is ~ 150 bp. We have found some manually confirmed cases of DTM63 insertion in promoter regions, which indicate DTM63 potential role in gene structure variations and may regulate downstream gene expression (Additional file 4: Figure S4; homologous regions listed in Additional file 7: Table S3). In our previous study we reported that MITE insertion is involved in emergence of nucleus polyembryony in *Citrus* [27], which also highlight the pivotal regulatory role of MITEs in promotor regions. In further studies, whether DTM63 insertion change downstream gene expression and even cause phenotype mutations in *Citrus* will be investigated.

### The number of complete LTR retrotransposons depends highly on genome integrity

We notice that the number of complete LTR retrotransposons highly depends on the genome integrity. For example, *C. grandis* was sequenced by Pacbio long reads (contig N50, 10.62 Mb) and *C. clementina* was sequenced using Sanger sequencing method (contig N50, 115.9 kb), while the other 4 genomes were sequenced and assembled using next-generation sequencing (NGS) approach (all their contig N50 < 80 kb). Therefore, the number of identified complete LTR retrotransposons in *C. grandis* is the highest, followed by *C. clementina*, while the other 4 genomes have less complete LTR retrotransposons. As the 5’LTR and 3′ LTR of complete LTR retrotransposons, especially newly inserted LTR retrotransposons should be identical, but in this case, NGS assembly software tend to disrupt the 5’LTR and 3’LTR into different contigs, while long reads spanning the 5′ /3’LTR with flanking sequence usually overcome this biasness. The length of MITEs is relatively short (usually < 800 bp), therefore the number of full-length MITEs is basically unaffected by genome integrity.

### Whole genome comparison reveals the role of LTR retrotransposons in genome diversity

Our whole genome LTRs annotation and comparison give insights into the role of different LTR retrotransposons families in genomes diversity. Some LTR retrotransposons are less activated with few new solo-LTR formation in recent years and contributed less diversity among different *Citrus* genomes, while some families are highly divergent among different *Citrus* genomes and may play a part in *Citrus* genome diversity. As we highlighted above, the number of complete LTR retrotransposons highly depends on genome integrity, further studies on more complete *Citrus* genomes will be needed to validate whether complete LTR retrotransposons shows same patterns of solo-LTRs. Although an insertion of *Copia*-like retrotransposon upstream of Ruby (a MYB transcriptional activator) was identified and controlled the expression of Ruby [20], which further reveals the regulatory role of LTR insertions for downstream genes. It remains to be seen whether other LTRs insertions show similar effect and even impact the phenotype of *Citrus* or not.

## Conclusions

In this study, we focused on MITEs and LTR retrotransposons in 6 *Citrus* species and found that MITE-related sequences accounted for about 3% of each genome, while LTR retrotransposons accounted for about 21.2% to ~ 33.1%. One-round amplification dominates the amplification mode of MITE families with high-copy numbers. Among these, DTM63 was possibly the first discovered active *Mutator*-like MITE family in *Citrus*. 12.9% of the small RNAs in sweet orange were derived from MITE-related sequences, indicating an important regulatory role of MITEs. The insertion of MITEs in the *Citrus* genome led much polymorphism, and the inter-genus ratio of MIP is much higher than intra-genus, which reflects the genetic relationships. Moreover, by comparing the LTR retrotransposons content on chromosomes, we found that LTRs predominantly enrich in centromere-proximal region, and Solo LTRs formation has a positive relation with LTR insertion time, LTR size and the ratio of LTR/INTERNAL. The insertion and elimination of LTR-RTs accomplished by a dynamic balance in *Citrus* genomes, and the half-life of LTR-RTs is longer than *Arabidopsis*, Rice and *Medicago truncatula*. These findings provide insights into MITEs and LTR retrotransposons and their roles in genome diversity in different *Citrus* species genomes.

## Methods

### Genomic sequences

*C. sinensis, A. buxifolia*, *C. grandis*, *C. ichangensis, and C. medica* were sequenced by us [27] and available at *C. sinensis* annotation project database (http://citrus.hzau.edu.cn/orange/). While, the genome of *C. clementine* was downloaded from *Citrus* Genome Database (https://www.citrusgenomedb.org/).

### MITE annotation, manual curation and classification

MITE-Hunter [31] (default parameters) was used to search all MITE candidates from the selected genomes followed by UCLUST, identity 80% was used to cluster the candidates. We picked at least 1 representative from each cluster to perform manual correction for TSD and terminal inverted repeats (TIR). We removed the false-positive sequences which did not have TSD and TIR features, and then removed the sequences with length greater than 800 bp to keepF41 in line with the previous studies. Since some of the sequences have changed, we perform the second clustering (family) with the same parameters. MITEs superfamily classification was referred to a previous study [42]. Finally, we manually picked one MITE with complete TIR and TSD as representative from each family to construct *Citrus* MITE database. RepeatMasker 4.0.2 (parameters “-pa 6 -s -nolow –xsmall -excln”) [43] was used to annotate MITE-related sequences for all 6 selected genomes, and an in-house Perl script was written to retrieve all MITE-related sequences (Additional file 1: Figure S1).

### MITE amplification mode and time

MITE-related sequences which cover over 90% of the representatives were treated as full-length MITEs. MUSCLE [44] was used to align the full-length MITEs which belong to the same family, and then MEGA6 [45] was used to construct phylogenetic tree with neighbor-joining method. According to the Jukes-Cantor method [46], we wrote a Perl script to calculate the pairwise divergence of each MITE family. Using kiwifruit’s average substitution rate of 2.81 × 10^− 9^ substitutions per synonymous site per year in coding region [47] and 2 times the rate in non-coding region (*r* = 5.62 × 10^− 9^), and formula T = k/2r [13], we estimated the amplification time of several MITE families with high-copy number.

Putative *Mutator*-liker DDE regions in *C. sinensis* and *C. clementina* were obtained with TARGeT (*e* value<=0.01) pipeline [48]. Flanking sequences with 10 kb upstream and downstream of the putative region were retrieved. A Perl script was written to find the conserved terminal sequences of 5′-GGACTTG-3′, 5′-CAGGTCC-3′ (allowing 1 mismatch in the terminals), and 9~10 bp TSD in all candidates.

### P/A polymorphism of MITEs

Flanking sequences with 1 kb upstream and downstream of the full-length MITEs were retrieved from all six genomes. First, the flanking sequences of MITE from the other five genomes were aligned to sweet orange genome using BLAST (*e* value< 10^− 50^). Then the flanking sequences of sweet orange were aligned to the other five genomes. If the pair of flanking sequences was the best hits and anchored to the same scaffold/chromosome, same strand, and the distance between the two anchored sites was less than 1 kb, the MITE loci were thought to be allelic. If there was a MITE-related sequence of the same MITE family in the above target allelic loci, the MITE insertion was thought to be present in both genomes, otherwise the MITE insertion was thought to be specific for the query genomes and absent from the target genome. A common MITE locus index was created based on the pairwise relation between *C. sinensis* and the other five *Citrus* species to analyze its distribution and role across all the six *Citrus* genomes. We defined the first 1 kb sequence region of the gene transcription start site as the promoter region. Bedtools [49] v2–2.26.0 was used to identify the insertion/presence of MITEs in different genome regions (5’UTR, 3’UTR, intron, intergenic, promoter). DREME tool of the MEME Suite (http://meme-suite.org/tools/dreme) was used for MOTIF analysis. Several in-house PERL scripts were written to measure relative abundance density of MITEs in *Citrus* genomes.

### MITE-derived small RNAs

In order to prevent deviation of relative position statistics caused by incomplete MITE, the length of all the MITE’s sequences with the difference of less than 1% of the length of the seed sequence was selected from each family. BOWTIE2 [50] allowing for 1 base mismatch was used to compare small RNAs came from previous studies to the selected MITE’s sequences. Small RNAs are believed to be derived from the MITE if they significantly aligned against MITE.

### LTR retrotransposons annotation and classification

tRNAScan-SE [51] was used to predict the tRNAs of 6 species with default parameter, and then all tRNAs were combined after filtering pseudogenes and redundant sequences to make a *Citrus* tRNA database. The tRNA data was used to predict the PBS of LTR retrotransposons with LTR_FINDER [52] by using developed tRNA database. LTR_FINDER parameter “-w 2” was used to predict LTR retrotransposons. Similarly, as performed in a previous study [53], clustering and filtering was performed to reduce false positives, only candidates with 5’TG, 5’CA, PBS, PPT, 3’CA and 3’TG were considered in the further analyses. The annotation work flow for LTR is shown step by step in Additional file 2: Figure S2.

### LTR retrotransposons phylogenetic tree

Three frame translation of INTERNAL sequences was employed, and hmmsearch (e value ≤10^− 6^) was used to search the profile of *Gypsy*-like RT (PF00078) and *Copia*-like RT (PF07727). RT domain amino acid sequences longer than 100 aa were retrieved from the translation. We chose one typical RT sequence from each family, and premature stop codons were eliminated from the sequences. Combined with the representatives we collected from the previous studies, Muscle was used to align the RT sequences. MEGA6 [45] (default parameters) was used to construct phylogenetic tree with neighbor-joining method.

### The distribution of LTR retrotransposons on chromosomes

Using the above LTR annotation results and taking the sweet orange as reference example, chromosomes were split into 1 Mb windows, and the windows < 1 Mb for each chromosome were not taken into account in further analyses. The distribution of the LTR on the chromosome is counted.

### LTR insertion time

As in the transposition process, the newly generated LTR retrotransposon will only take one of the LTRs as template. In this way, the 5’LTR and 3’LTR of the newly generated LTR retrotransposon will be identical. During evolutionary time, 5’LTR and 3’LTR may accumulate spontaneous mutations. LTR insertion time (T) formula T = k/2r, using a substitution rate of *r* = 5.62 × 10^− 9^ substitutions per site per year for calculations. MUSCLE was used to align the 5′-LTR and 3′-LTR of each complete LTR retrotransposon and the distance between LTRs was corrected by the Jukes-Cantor method [46].

In addition, we inferred the insertion time of LTR in an exponential distribution. By using R language curve fitting nonlinear least squares (NLS) function (fitting equation y = a ∗ 2^*b* ∗ *x*^), initial value a = 270, b = − 0.5 and the exponential function, we explained the number and time of LTR insertion. Furthermore, for the LTR family with higher copy number in the *Citrus* species, the insertion time of each member was calculated separately, and then the family member amplification time was mapped.

### Solo-LTR formation

*C. grandis* was sequenced with the PacBio sequencing technology, so we chose *C. grandis* as the representative for solo-LTR formation analysis. Families with less than 10 complete LTR retrotransposons were not included in this study, as mis-annotation and omission of some family members are unavoidable, and families with more complete LTR retrotransposons would be more robust. As solo-LTRs only have one LTR, the methods used for complete LTR retrotransposons are not feasible for solo-LTRs studies. Therefore, we used the average insertion time of complete LTR retrotransposons of each family to represent the insertion time of solo-LTRs. The average INTERNAL sequence length was also calculated. The calculation of Spearman’s relationship between S/C and LTR insertion time, LTR size and LTR/INTERNAL were done using R software.

## Additional files

Additional file 1: **Figure S1.** The pipeline of MITE classification and annotation. (DOCX 151 kb)

Additional file 2: **Figure S2.** The pipeline of LTR retrotransposons classification and annotation. (DOCX 475 kb)

Additional file 3: **Figure S3.** Relative abundance of MITE families in *Citrus* species. (DOCX 1605 kb)

Additional file 4: **Figure S4.** Manually confirmed DTM63 insertion sites. (A) Manually confirmed case of *C. grandis.* (B). Manually confirmed case of *C. sinensis.* (C) Manually confirmed case of *C. clementina*. (D) Manually confirmed case of *C. ichngensis*. Sequences in blue represent TSD and the “N” in red represent a copy full-length DTM63. The corresponding homologous regions were listed in Additional file 7: Table S3. (DOCX 584 kb)

Additional file 5: **Table S1.** Information of conserved and non-conserved MITEs insertion between different genomic regions (DOCX 12 kb)

Additional file 6: **Table S2.** Information of newly inserted MITEs in different genomic regions of three *Citrus* species. (DOCX 13 kb)

Additional file 7: **Table S3.** Information of DTM63 insertion sites. (DOCX 14 kb)

Additional file 8: **Data Set S1.** The seed file of MITEs. (FA 43 kb)

Additional file 9: **Data Set S2.** The seed file of LTR retrotransposons. (FA 2229 kb)

## Abbreviations

LTR: Long terminal repeat
MITEs: Miniature inverted–repeat transposable elements
RE: Reverse transcriptase
sRNAs: Small RNAs
TSD: Target site duplication
TSS: Transcription start sites
TTS: Transcription termination sites
