# Supplementary figures and images for: Comparative analysis of miniature inverted–repeat transposable elements (MITEs) and long terminal repeat (LTR) retrotransposons in six Citrus species
Source: BMC Plant Biol. 2019 Apr 15;19:140. doi: 10.1186/s12870-019-1757-3 (PMC6466647; doi:10.1186/s12870-019-1757-3)

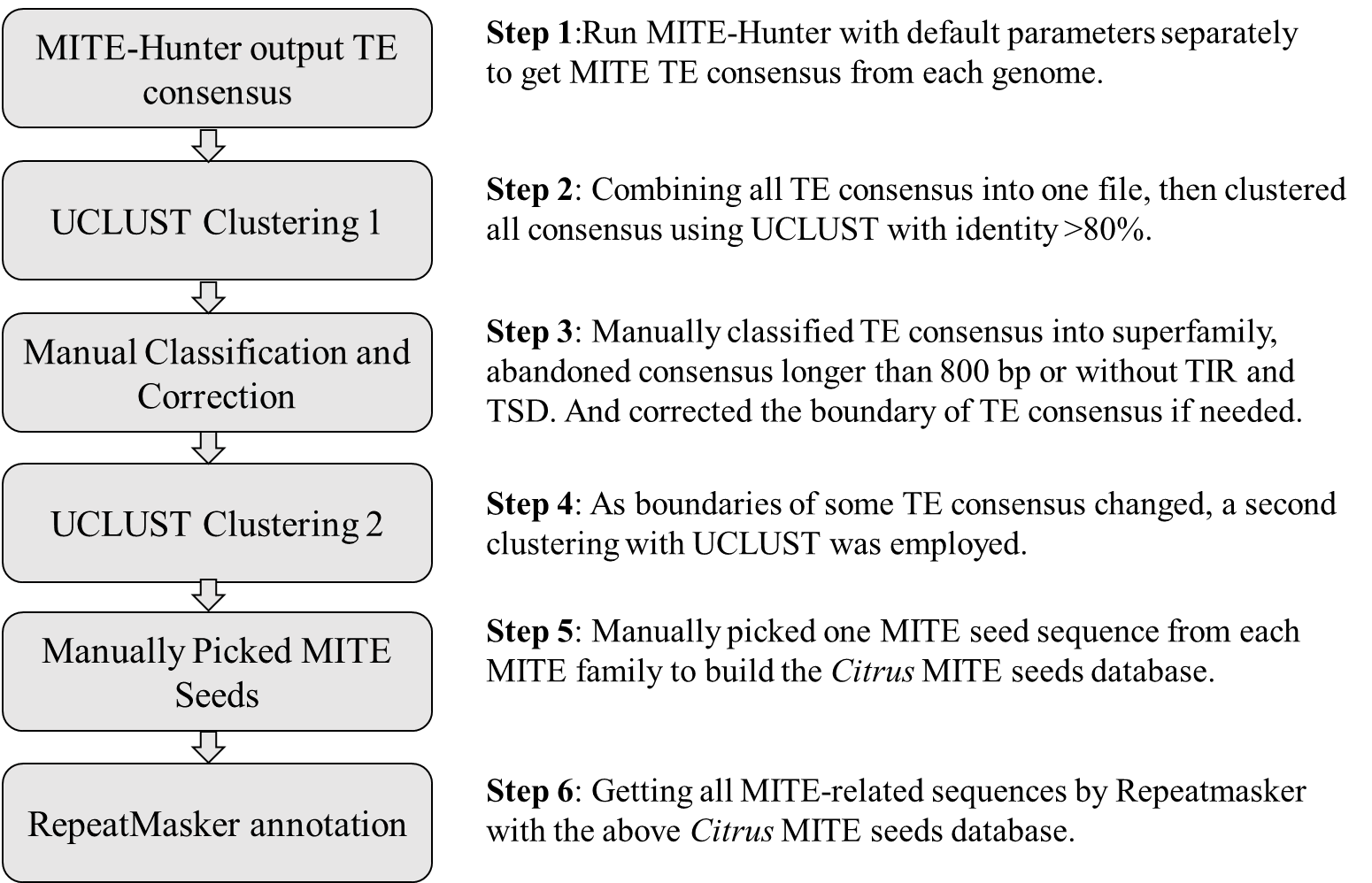


**Figure S1**. The pipeline of MITE classification and annotation.

Supplement: Supplementary file 1 — Figure S1. The pipeline of MITE classification and annotation. (DOCX 151 kb) [file 12870_2019_1757_MOESM1_ESM.docx]

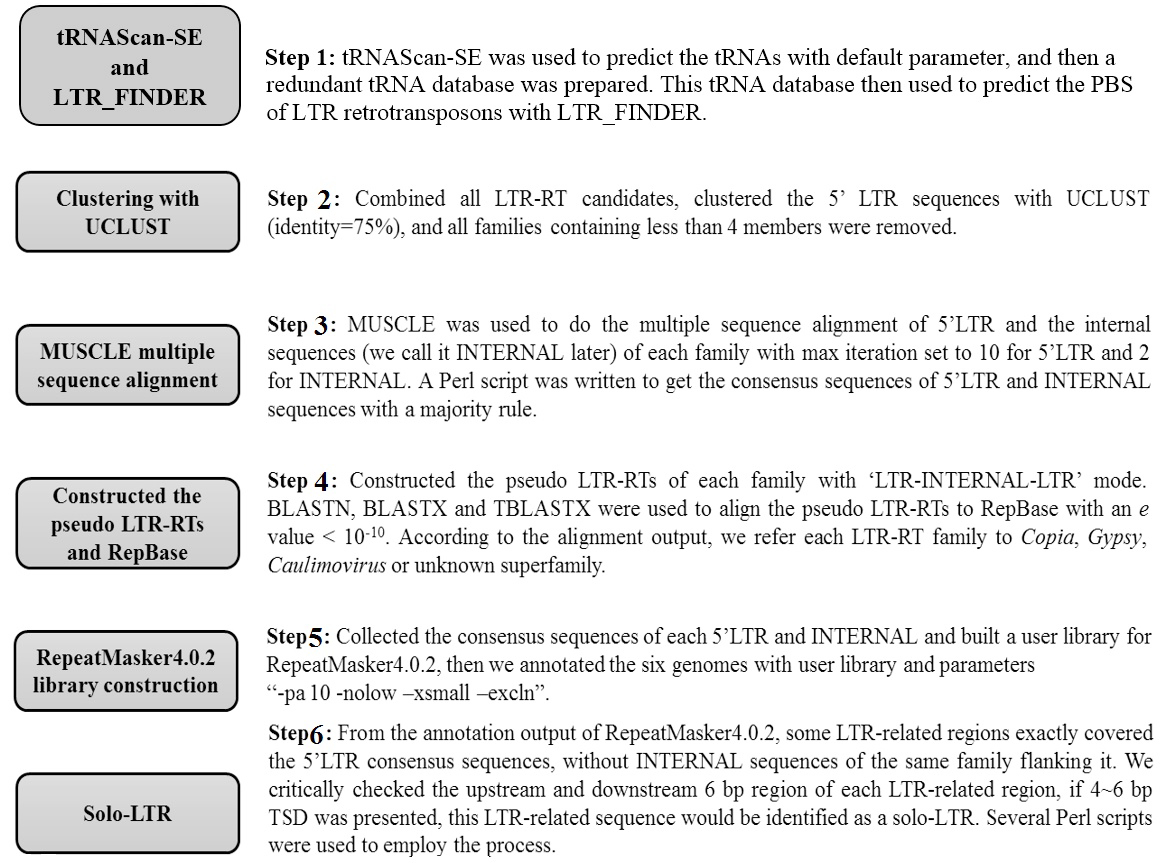


**Figure S2**. The pipeline of LTR retrotransposons classification and annotation.

Supplement: Supplementary file 2 — Figure S2. The pipeline of LTR retrotransposons classification and annotation. (DOCX 475 kb) [file 12870_2019_1757_MOESM2_ESM.docx]

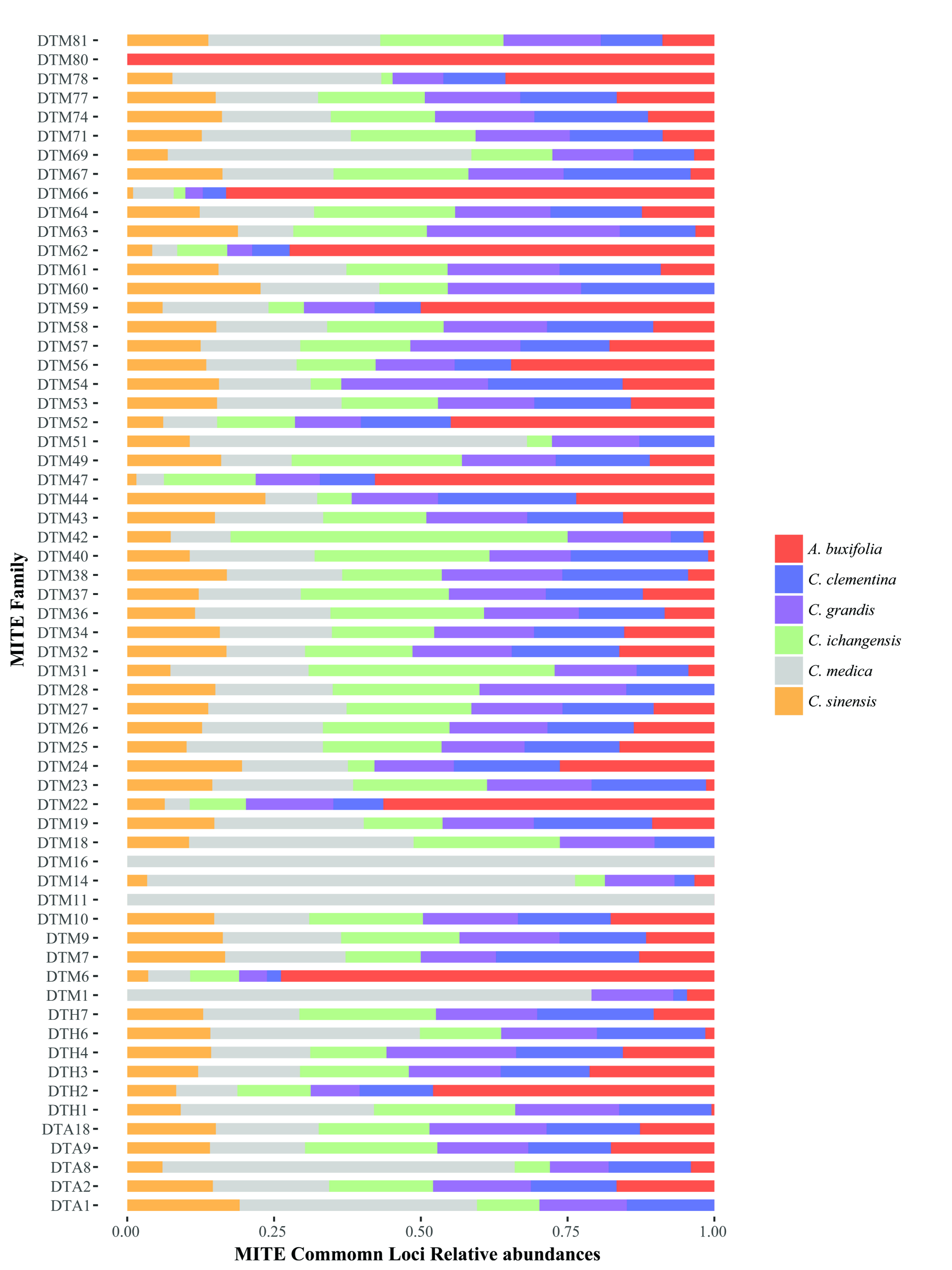


**Figure S3.** Relative abundance of MITE families in *Citrus* species.

Supplement: Supplementary file 3 — Figure S3. Relative abundance of MITE families in Citrus species. (DOCX 1605 kb) [file 12870_2019_1757_MOESM3_ESM.docx]
