# Supplementary material for: Comparative analysis of miniature inverted–repeat transposable elements (MITEs) and long terminal repeat (LTR) retrotransposons in six Citrus species
Source: BMC Plant Biol. 2019 Apr 15;19:140. doi: 10.1186/s12870-019-1757-3 (PMC6466647; doi:10.1186/s12870-019-1757-3)
Supplement: Supplementary file 4 — Figure S4. Manually confirmed DTM63 insertion sites. (A) Manually confirmed case of C. grandis. (B). Manually confirmed case of C. sinensis. (C) Manually confirmed case of C. clementina. (D) Manually confirmed case of C. ichngensis. Sequences in blue represent TSD and the “N” in red represent a copy full-length DTM63. The corresponding homologous regions were listed in Additional file 7: Table S3. (DOCX 584 kb) [file 12870_2019_1757_MOESM4_ESM.docx]

**A**

**
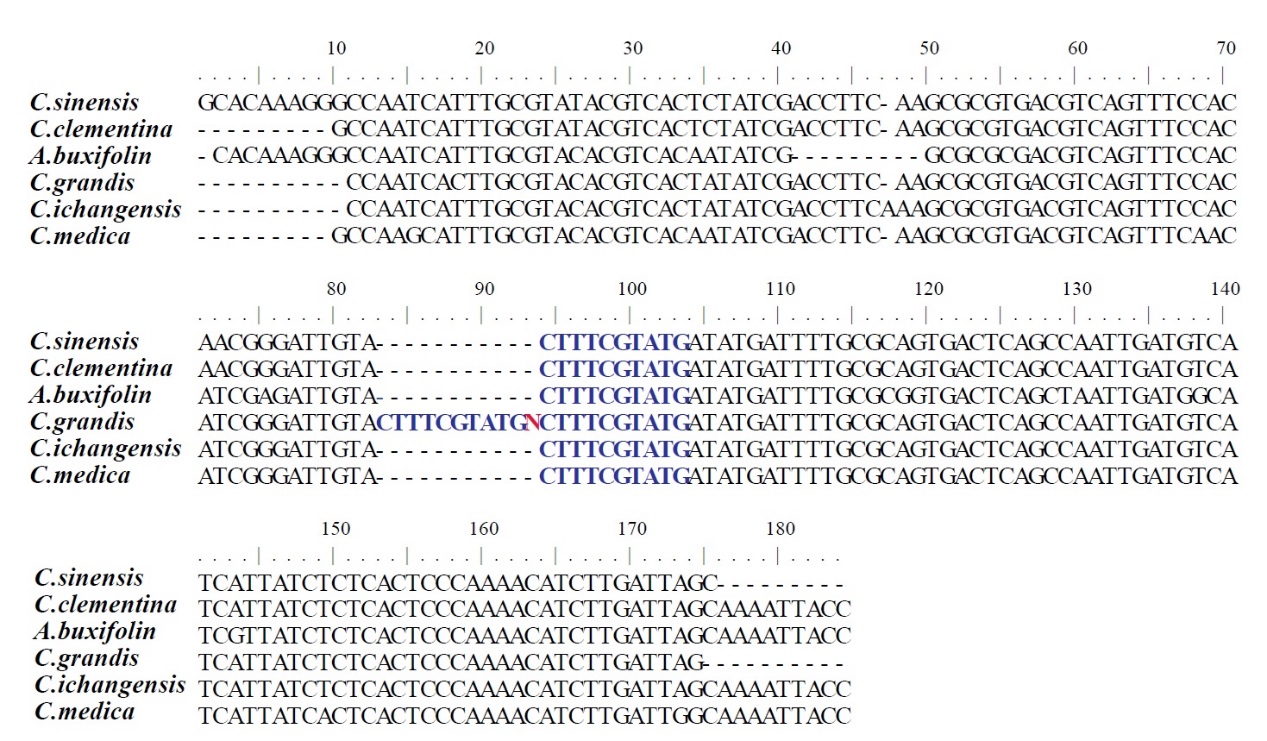
**

**B**


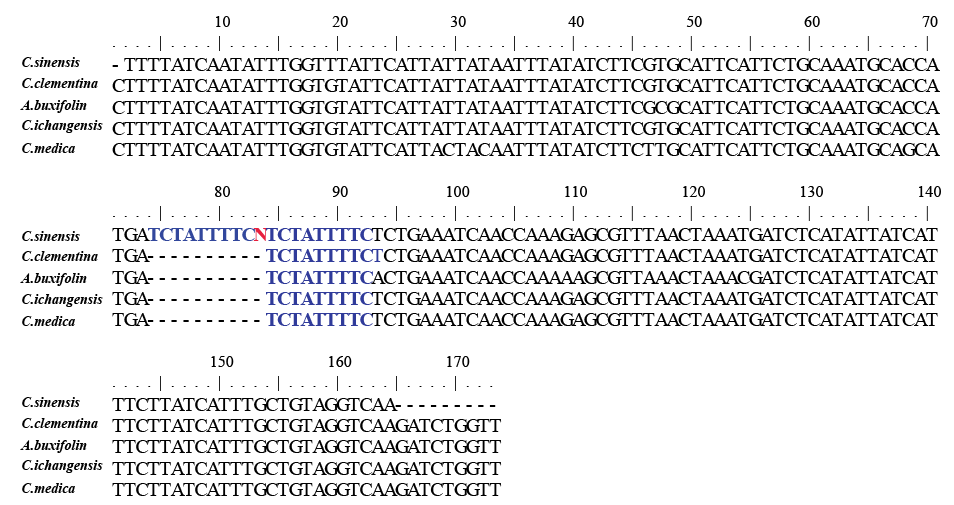


**C**


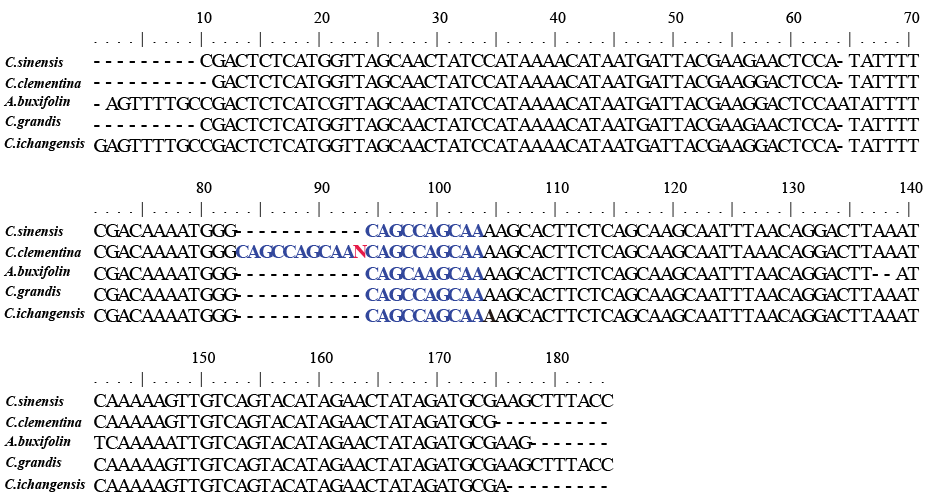


**D**


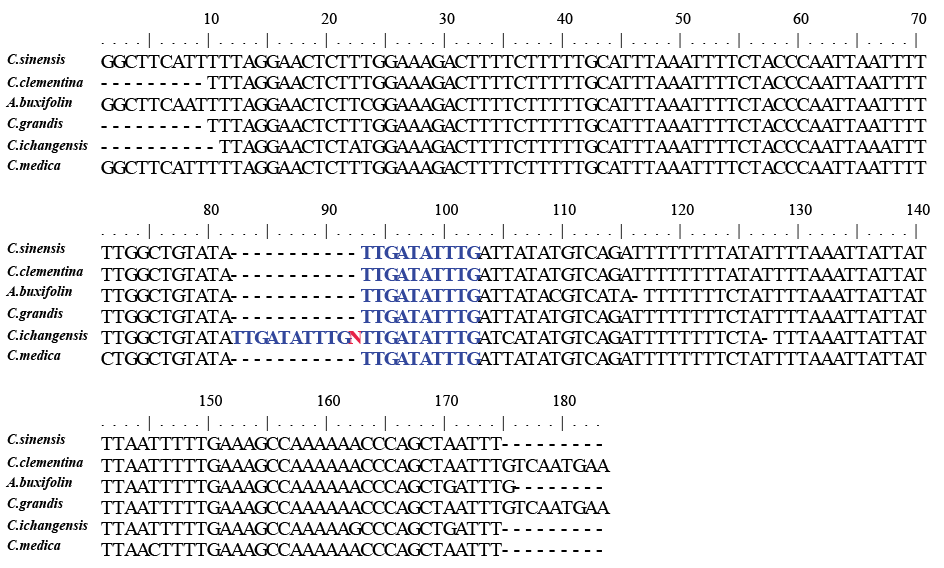


**Figure S4.** Manually confirmed DTM63 insertion sites. (A) Manually confirmed case of *C. grandis.* (B). Manually confirmed case of *C. sinensis.* (C) Manually confirmed case of *C. clementina*. (D) Manually confirmed case of *C. ichngensis*. Sequences in blue represent TSD and the “N” in red represent a copy full-length DTM63. The corresponding homologous regions were listed in Supplemental Table S3.
