# Supplementary material for: Comparative analysis of miniature inverted–repeat transposable elements (MITEs) and long terminal repeat (LTR) retrotransposons in six Citrus species
Source: BMC Plant Biol. 2019 Apr 15;19:140. doi: 10.1186/s12870-019-1757-3 (PMC6466647; doi:10.1186/s12870-019-1757-3)
Supplement: Supplementary file 5 — Table S1. Information of conserved and non-conserved MITEs insertion between different genomic regions (DOCX 12 kb) [file 12870_2019_1757_MOESM5_ESM.docx]

| **Table S1.** Information of conserved and non-conserved MITEs insertion between different genomic regions | | | | | |
| --- | --- | --- | --- | --- | --- |
|  | Promoter | Intron | 5‘UTR | 3‘UTR | Intergenic |
| Conserved loci | 18.13% | 16.60% | 0.19% | 1.53% | 63.55% |
| Non-conserved loci | 25.76% | 8.67% | 1.74% | 2.57% | 60.46% |
