# Supplementary material for: Comparative analysis of miniature inverted–repeat transposable elements (MITEs) and long terminal repeat (LTR) retrotransposons in six Citrus species
Source: BMC Plant Biol. 2019 Apr 15;19:140. doi: 10.1186/s12870-019-1757-3 (PMC6466647; doi:10.1186/s12870-019-1757-3)
Supplement: Supplementary file 6 — Table S2. Information of newly inserted MITEs in different genomic regions of three Citrus species. (DOCX 13 kb) [file 12870_2019_1757_MOESM6_ESM.docx]

| **Table S2.** Information of newly inserted MITEs in different genomic regions of three *Citrus* species | | | | | |
| --- | --- | --- | --- | --- | --- |
|  | Promoter | Intron | 5‘UTR | 3‘UTR | Intergenic |
| *C. clementina* | 26.08% | 9.48% | 0.22% | 1.29% | 62.72% |
| *C. sinensis* | 27.17% | 11.56% | 0.00% | 1.16% | 59.54% |
| *C. grandis* | 26.63% | 10.89% | 0.30% | 1.06% | 60.67% |
