# Supplementary material for: Comparative analysis of miniature inverted–repeat transposable elements (MITEs) and long terminal repeat (LTR) retrotransposons in six Citrus species
Source: BMC Plant Biol. 2019 Apr 15;19:140. doi: 10.1186/s12870-019-1757-3 (PMC6466647; doi:10.1186/s12870-019-1757-3)
Supplement: Supplementary file 7 — Table S3. Information of DTM63 insertion sites. (DOCX 14 kb) [file 12870_2019_1757_MOESM7_ESM.docx]

| **Supplemental Table S3.** Information of DTM63 insertion sites | | | | |
| --- | --- | --- | --- | --- |
| **Species** | **Data in Fig. S4A** | **Data in Fig.S4B** | **Data in Fig.S4C** | **Data in Fig.S4D** |
| *Atalantia buxifolia* | scaffold5535 2517818-2517980 | scaffold29305  74967-75129 | scaffold18357  249026-249188 | scaffold26433  1319481-1319643 |
| *Citrus sinensis* | chrUn  4321675-4321837 | chr2  487865-488178 | chr5  14402237-14402399 | chr7  24158417-24158579 |
| *Citrus clementina* | scaffold_3  49852891-49853053 | scaffold_2  36179166-36179328 | scaffold_3  11138924-11139237 | scaffold_4  6970277-6970439 |
| *Citrus medica* | scaffold_138  481350-481512 | scaffold_333  131708-131870 | NA^#^ | scaffold_199  196703-196865 |
| *Citrus ichangensis* | scaffold_1  1679796-1679958 | scaffold_140  202215-202377 | scaffold_183  246632-246794 | scaffold_21  670179-670492 |
| *Citrus grandis* | chr5  48167397-48167710 | NA^#^ | chr5  18550148-18550310 | chr7  6210761-6210923 |
|  |  |  |  |  |

# indicates that no homolog region was found in that species.
